# Early longitudinal experiences increase medical student self-efficacy

**DOI:** 10.15694/mep.2021.000142.1

**Published:** 2021-05-24

**Authors:** Kanwarabijit Thind, Patrick Bauer, Hilary Titus, Meredith Niess, Reem Hasan

**Affiliations:** 1 Oregon Health & Science University; 2 University of Utah; 3 Charlotte Community Health Clinic

**Keywords:** self-efficacy, patient-centeredness, longitudinal experiences, early medical education, preceptorship

## Abstract

This article was migrated. The article was marked as recommended.

**Background:** The purpose of this study was to investigate the impact of early longitudinal preceptorship experiences on the evolution of general self-efficacy and patient-centeredness self-efficacy through medical school.

**Methods:**Validated surveys for general self-efficacy and patient-centeredness self-efficacy were administered in an allopathic medical school at three timepoints. These data were stratified by traditional and longitudinal preceptorship groups and analyzed using Generalized Estimating Equations. Qualitative analysis of narrative medicine essays by the same cohort during their preceptorship experiences was also performed.

**Results:** While general self-efficacy remained largely unchanged over time, patient-centeredness self-efficacy measures increased throughout medical school in the whole cohort (N=157). The longitudinal preceptorship group had higher gains in patient-centeredness self-efficacy, especially in the domain of exploring patient perspectives (p<0.05). The qualitative analysis of narrative medicine essays showed those in longitudinal preceptorships were more likely to discuss health care systems issues, consider psychosocial factors, and perceive themselves as active members of the care team.

**Discussion:**Our study indicates greater patient-centeredness attitudes over time among students who have early longitudinal experiences, perhaps due to the self-efficacy building nature of these experiences. Our research suggest that medical school programs should consider incorporating opportunities for early longitudinal clinical experiences for their students.

## Introduction

General self-efficacy is a concept long studied in psychology that represents one’s judgement of their own ability to complete the actions necessary to deal with a variety of prospective situations ( Artino, 2012; Bandura, 1977). This concept has expanded to many fields, including extensive use in medical education ( Artino, 2012; Mavis, 2001). As medical educators work to support students in medical training and performance, self-efficacy has emerged as an important domain for interventions ( Stegers-Jager, Cohen-Schotanus and Themmen, 2012; Klassen and Klassen, 2018).

Patient-centeredness encompasses several qualities focusing on the patient’s individual needs, preferences, and context with three core attributes: a) consideration of patients’ needs, perspectives, and individual experiences, b) provision of opportunities to patients to participate in their care, and c) enhancement of the patient-clinician relationship ( Epstein *et al.,* 2005). The literature shows the importance of patient-centered principles in delivering high quality healthcare, increasing patient satisfaction, and alleviating physician burnout ( Shanafelt, 2009).

Through self-efficacy theory, students with increased perceived “general self-efficacy” and “patient-centeredness self-efficacy” are hypothesized to have higher academic and career performance ( Bandura, 1977). It is unclear how either general or patient-centeredness self-efficacy changes throughout medical school and residency training, how competing pressures (i.e. high-stakes tests like USMLE Step tests, family obligations, etc.) impacts these trends, or if increased clinical exposure leads to more accurate self-efficacy assessment.

Medical education is shifting to facilitate meaningful, professional roles for students early in their education ( Curry, 2014; Smith *et al.,* 2013). This has been attempted through strategies such as student-run clinics ( Schutte *et al.,* 2015), community-based service learning ( Hunt, Bonham and Jones, 2011), and longitudinal clinical experiences ( Hauer *et al.,* 2012). Early substantial contributions increase medical student knowledge and skills, and build a sense of professional identity ( Smith *et al.,* 2013). A qualitative study examining the results of a longitudinal preceptorship in primary care suggested that student skills improved by way of the evolution of the faculty-student-patient relationships over time ( Walters *et al.,* 2011). Further research is indicated to understand how early longitudinal student-patient relationships impact student outcomes, particularly through validated methods ( Vijn *et al.,* 2017).

We examine how longitudinal preceptorship experiences during the first year of medical school impacts student outcomes. Early longitudinal preceptorship experiences may be protective against any loss of self-efficacy and patient-centered behavior through many factors such as increased strength of preceptor-student relationship, continuity of student cohort peers, and promotion of small group discussion of clinical experiences.

The objectives of this study include: A) To report the evolution of general self-efficacy and patient-centeredness self-efficacy during medical school; B) To outline the impact of early longitudinal experiences on self-efficacy in a medical student cohort using qualitative thematic analysis of narrative medicine essays and quantitative surveys.

## Methods

### Participants

Two groups of students were followed over four years in a single medical school cohort: (1) traditional preceptorship students and (2) longitudinal preceptorship students. Students in the traditional preceptorship group rotated through three, 8-week experiences with three physicians in any specialty during the pre-clinical medical school years. Examples of specialties where students rotated in the traditional preceptorship include neuroradiology, medical intensive care unit, general hospitalist, pediatrics, community family medicine, general surgery, trauma surgery, vascular surgery, and urology among many others. Students observed the physicians and practice settings to learn more about professionalism, interpersonal skills and communication, practice-based improvement, and interprofessional teamwork.

Students in the longitudinal preceptorship group participated in one of two types of experiences. (1) The Student Navigator Project (SNaP), an 18-month pre-clinical patient navigation program with a focus on health systems science. Students served as patient navigators for complex patients in primary care clinics to learn about health system barriers and the community context of care. They also developed basic clinical skills and interprofessional teamwork skills by working alongside medical assistants (MA) in clinics. Students completed a mentored group improvement project in their clinic, supplemented by improvement science didactics. Lastly, students practiced evidence-based medicine presentations and completed a communication curriculum on trauma-informed care, motivational interviewing, and health coaching ( Hasan *et al.,* 2020). (2) The longitudinal family medicine preceptorship in which students were paired with one family medicine provider for 10 months in the pre-clinical years of medical school. Students worked with the provider weekly to develop history taking and physical exam skills through continuity of patients, clinical site and provider/mentor. For the purposes of this report, both of these preceptor models are considered ‘longitudinal.’

### Quantitative measures

General self-efficacy was measured using the General Perceived Self-Efficacy Survey (PSE), a 10-item, validated survey that has been used in a variety of fields ( Luszczynska, Scholz and Schwarzer, 2005). The Self-efficacy in Patient-Centeredness Questionnaire (SEPCQ-27) was also used to understand students’ perceived ability to perform key patient-centeredness elements. The validated 27-item survey was subdivided by the creators into three underlying self-efficacy factors using component analysis: 1) Exploring the patient perspective, 2) Sharing information and power, and 3) Dealing with communicative challenges ( Zachariae *et al.,* 2015).

### Design

Both surveys were administered to the class cohort at three, unequally spaced times: (1) In the Winter of their first year of medical school (Baseline; before any meaningful clinical experience during preceptorship), (2) In the Transitions to Clinical Experience (TTCE) course (1.5 years into medical school, just after completing USMLE Step 1), and (3) In their fourth year during the Transitions to Residency (TTR) course (just prior to graduation). A visual representation of this timeline can be found in Appendix A. Surveys were voluntary, and participant information was linked across surveys.

Additionally, students completed narrative medicine essays during their first year of medical school. Essay topics focused on development of self-efficacy and patient-centeredness. Two groups of students (from the same cohort who participated in the surveys) were examined, with 6 students randomly selected from each group: (1) students who participated in traditional preceptorships and (2) students who participated in one of the longitudinal preceptorships. Four essays were examined from each of the 12 students (n=48 essays).

### Data analysis

Quantitative survey analysis was performed using Stata Version 12 (StataCorp, College Station, Texas, USA). Basic descriptive statistics were calculated to understand changes in survey scores over time. We used Generalized Estimating Equations (GEE) to model the change in score over time in medical school, adjusting for type of preceptorship experience. GEE allowed us to model clustering of scores by individual, and account for unequal cluster sizes and missing data (as response rates differed by survey). We used robust standard errors and an unstructured correlation matrix as this makes no assumptions about the underlying structure of the correlation matrix. Given the small number of repeated measurements per individual respondent, this was appropriate for our model. We graphed the data to ensure model assumptions were met.

Narrative medicine essays were examined using thematic analysis with ATLAS.ti Version 8.0 (ATLAS.ti Scientific Software Development GmbH, Berlin, Germany). Two blinded researchers completed dual coding, with a third coder performing reconciliation of discrepancies. Example of the codebook used can be found in Supplementary File 1.

IRB deemed this study exempt from oversight (OHSU IRB #00016160).

## Results/Analysis

### Survey Respondents

Of the 157 first year medical students, 136 were in traditional preceptorships and 21 were in longitudinal preceptorships. Response rates are outlined in Table 1. At the baseline survey, 134 traditional (98.53%) and 21 (100%) longitudinal preceptorship students responded. During TTCE, 43 traditional (31.62%) students and 8 longitudinal (38.10%) students responded. During TTR, 35 traditional (25.74%) and 6 longitudinal (28.57%) students responded. The survey form combined the two surveys with the PSE survey occurring first. However, some respondents only filled out the PSE survey ( Table 1). There were similar response rates for all time points between the traditional and longitudinal preceptorship groups. However, the longitudinal group responded at a slightly higher rate for some survey time points.

**Table 1:** Description of respondents per survey in overall cohort

Survey Time Points	Overall Cohort (N=157)	%	Traditional Preceptorship Group (N=136)	%	Longitudinal Preceptorship Group (N=21)	%
Either Survey						
Baseline	155	98.73%	134	98.53%	21	100.0%
TTCE *	51	32.48%	43	31.62%	8	38.10%
TTR *	41	26.11%	35	25.74%	6	28.57%
SEPCQ-27 Survey Only						
Baseline	110	70.06%	95	69.85%	15	71.43%
TTCE	46	29.30%	40	29.41%	6	28.57%
TTR	38	24.20%	33	24.26%	5	23.81%
PSE Survey Only						
Baseline	144	91.72%	124	91.18%	20	95.24%
TTCE	51	32.48%	43	31.62%	8	38.10%
TTR	41	26.11%	35	25.74%	6	28.57%

* TTCE: Transitions to Clinical Experience; TTR: Transitions to Residency

**Figure 1:**
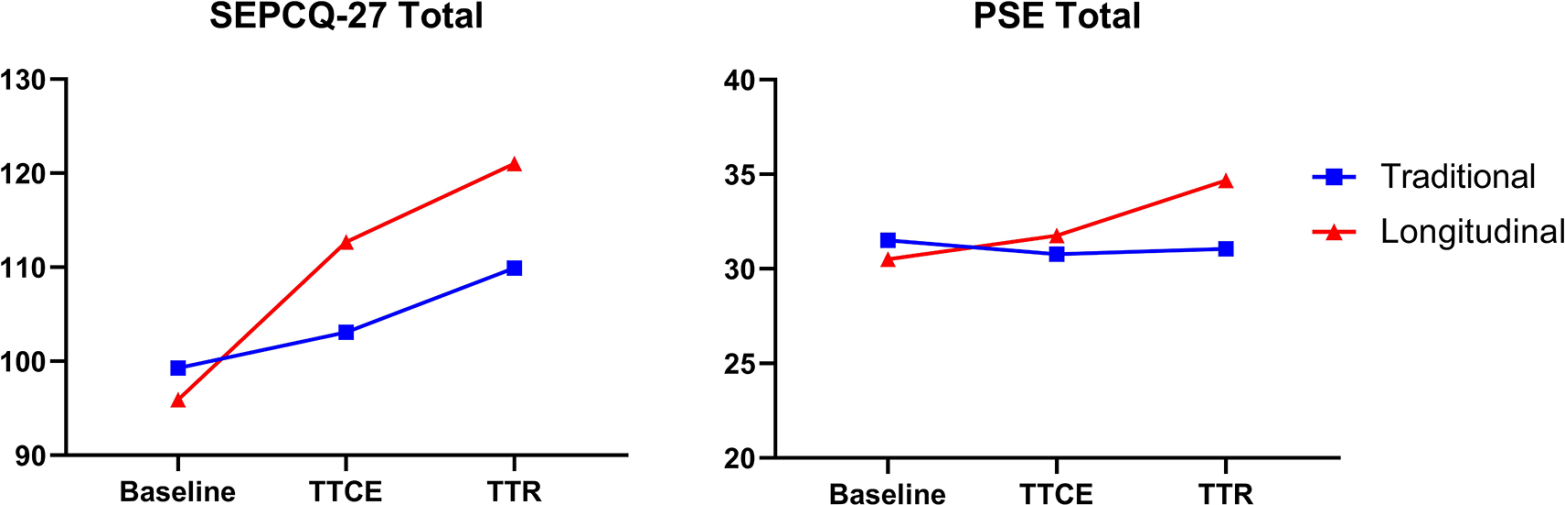
Mean profile plots of SEPCQ-27 and PSE survey scores through three timepoints, stratified by preceptorship type

The SEPCQ-27 survey results are reported both in aggregate and sub-divided into three sections representing different factors of patient-centeredness self-efficacy. The SEPCQ-27 total and PSE total survey score means stratified by preceptorship type (traditional or longitudinal) are represented in Figure 1. The longitudinal and traditional preceptorship groups began medical school with similar self-efficacy profiles. Over the two subsequent time points, the overall cohort showed a consistent increase in both total SEPCQ-27 survey scores and all three of the component factors (similar to the traditional group represented in Figure 1). GEE modeling of the whole cohort showed significantly increasing coefficients when comparing TTCE and TTR to Baseline survey time points for the total SEPCQ-27, SEPCQ factor 1 (exploring patient perspectives), and SEPCQ factor 3 (dealing with communicative challenges). SEPCQ factor 2 (dealing with communicative challenges) only had significant coefficient when comparing the Baseline to TTR time points ( Table 2). The PSE survey results for the overall cohort appear stable throughout the three time points ( Figure 1) and the modeling showed that time did not correlate with any changes in PSE results ( Table 2).

The SEPCQ-27 (in total and subdivided into the three factors) and the PSE survey scores were stratified by preceptorship type. Both the traditional and longitudinal preceptorship groups showed increasing SEPCQ-27 total scores between the three time points, and the longitudinal group had additive increases beyond that experienced by the traditional group in SEPCQ-27 total score (p<0.05), and all domains for at least one time period ( Table 2). Factor 3 (dealing with communicative challenges) showed significant increases for the longitudinal group at both subsequent surveyed timepoints.

For the PSE survey, neither group showed differences in survey scores between the three time points. As seen in Figure 1, there was a stable trend in scores for the traditional group, with the longitudinal group showing a larger increase in mean score by the TTR survey time point. However, in the GEE modeling shown in Table 2, preceptorship type was not shown to correlate with any significant changes in PSE Total score (-0.9 CI: -2.3, 0.6). In examining the model interaction terms, the longitudinal group did demonstrate a significant increase in score at the final surveyed timepoint prior to graduation (p<0.05).

**Table 2:** Results from GEE modeling of the surveys based on preceptorship type and survey time point, including interaction terms

Variables	SEPCQ-27 Total Coeff. (CI)	SEPCQ Factor 1 Coeff. (CI)	SEPCQ Factor 2 Coeff. (CI)	SEPCQ Factor 3 Coeff. (CI)	PSE Total Coeff. (CI)
Longitudinal Preceptorship	3.4 (-10.2, 3.3)	-0.3 (-3.3, 2.7)	-1.8 (-5.2, 1.6)	-1.3 (-3.1, 0.4)	-0.9 (-2.3, 0.6)
Time: Baseline to TTCE	4.8 (0.4, 9.2)	2.3 (0.72, 3.8)	1.0 (-1.1, 3.1)	1.8 (0.4, 3.2)	-0.7 (-2.1, 0.7)
Time: Baseline to TTR	11.2 (6.7, 15.6)	2.9 (1.2, 4.6)	5.9 (4.0, 7.8)	2.3 (0.9, 3.6)	-0.6 (-1.5, 0.4)
Longitudinal#TTCE *	13.3 (2.7, 23.9)	2.8 (-2.1, 7.7)	5.7 (-1.0, 12.4)	4.5 (2.5, 6.4)	2.0 (-1.0, 5.1)
Longitudinal#TTR *	12.6 (4.6, 20.5)	4.6 (0.8, 8.4)	4.6 (0.04, 9.2)	3.4 (1.5, 5.3)	2.9 (0.5, 5.3)

* Longitudinal#TTCE and Longitudinal#TTR represent the interaction term of being in a longitudinal preceptorship and being in the TTCE or TTR survey timepoint, respectively.

### Narrative Medicine Data

Thematic analysis of narrative medicine essays on self-efficacy and patient-centeredness was performed ( Table 3). Two groups were compared: students in traditional (n=6) vs longitudinal preceptorships (n=6). Four themes emerged that highlighted differences and similarities between the two groups: (1) manifestations of self-doubt and self-confidence, (2) developing empathy by taking the patient’s viewpoint, (3) characterizations of the role of healthcare professionals, and (4) observation versus action.

Both groups discussed the role of pretending in their development of self-efficacy with various perspectives as to its benefit and harm. They also discussed the central role self-perception played, with all essays being coded for at least one instance of either self-doubt or self-confidence. Some key differences between the groups include that longitudinal students may have played a more active, value-added role in their patient encounters. They were more likely to discuss biopsychosocial issues related to their patients as well as engage in interactions with patients on their own or as the leaders of the conversation than were students from the traditional, non-longitudinal group.

**Table 3:** Summary of narrative medicine essay thematic analysis between two preceptorship groups

Traditional Preceptorship Students	Longitudinal Preceptorship Students
**Manifestations of self-doubt and self-confidence**	
“I am gaining a lot of knowledge and would not trade these preceptorship opportunities for other activities, but I still do not see how I am adding value to a patient’s care as the clinic runs just as smoothly without my questions, without my presence.”	“Thus far I believe the program has done a good job at allowing us to interact with the patients and making us feel integrated into the medical team. And I believe that once a health provider is a part of a team, it’s hard not to feel valuable”
**Developing empathy by taking the patient’s viewpoint**	
“I was judging the patient’s behavior, and...I was feeling defensive for the doctors, but I forgot to think for the patient and his family. I was not putting myself in his shoes.”	“Although she still seemed active physically, through regular walks, and mentally through helping with her husband’s business, I wondered how frustrating it was for the patient to have problems recalling certain information.”
**Characterization of the roles of healthcare professionals**	
“As a future physician, particularly in these complicated political times, I think the big part of my role is to advocate for my patient’s health and to help them navigate the health care system as a whole.”	“[O]ften I find physicians in training have this righting reflex, the desire to fix all the patient’s problems, but sometimes in complex patients, some or even most of their problems can’t be solved and that the best thing for these patients is taking the time to be heard by a medical professional who cares.”
**Observation vs action**	
“I feel like I am an observer in this whole process. I do not yet have enough knowledge to provide insights into diagnosis, disease progress, or care and treatment. I am not providing comfort to the patient or family.”	“Now, we are actually independently functioning as MAs under the supervision of an MA in the clinic and communicating with physicians through that role, which I have found extremely interesting.”

Overall, students between the two groups were more similar in responses than different, with a high level of individual variability on self-efficacy and patient-centeredness concepts. Longitudinal students were more likely to express nuanced understanding of the impact of health system structure on patients. They were more likely to discuss their role as a health care team member with examples of first-hand experience.

## Discussion

Early medical education marks the beginning of professional development in medicine. Early student experiences, particularly longitudinal and continuity experiences with preceptor role models, impact the development of self-efficacy and patient-centeredness behaviors. We found that overall, there is an increase in general self-efficacy and self-efficacy of patient-centeredness as the cohort moved through medical school.

Comparing to graduate residency education, a study examining a patient- and family-centered care curriculum in a pediatrics residency found no significant changes in the pre-/post- patient-centered attitude mean score when comparing before residency and 2 years into residency ( Mann *et al.,* 2013). This could indicate that as students’ progress through medical school programs, they gain experiences that develop their patient-centeredness skills and attitudes (as indicated by our study), which remain constant through further training. This suggests that early influences and interventions have a larger impact on patient-centeredness, perhaps due to students being more impressionable at early stages.

In particular, our data shows that the longitudinal preceptorship group had higher increases in patient-centeredness self-efficacy compared to the traditional group during medical school. Specific domains that showed strong associations focused on taking the patient perspective and communication skills. The narrative essays showed high individual variability, but indicate that students in the longitudinal preceptorship experiences do indicate increased discussion of larger systemic and social issues for their patients and indicate their efficacy as part of the care team.

This may suggest that early longitudinal patient care experiences may be most beneficial in understanding how to communicate early and become comfortable with the information and power relationship between providers and patients, as these skills are modeled and practiced in a safe setting with familiar patients.

Both the traditional and longitudinal groups had no appreciable changes in PSE scores after preceptorship or clerkships. However, the longitudinal group had a jump in mean score between baseline and TTR survey. A study looking at the self-efficacy of problem-based learning similarly found little difference in self-efficacy between second and third year medical students ( Demirören, Turan and Öztuna, 2016). This may indicate diminishing returns on self-efficacy with more experience, or more honest appraisal of self-efficacy as students gain more experience.

It is interesting that our data showed significant increases in patient-centeredness self-efficacy (a specific metric), but not in general self-efficacy. This suggests that student self-appraisal of self-efficacy is easier when examining specific skills like patient centeredness and communication rather than general self-efficacy.

Our study involves a one cohort of medical students from a single institution, with loss to follow up at each progressive time point. This leads to a higher error and wide confidence intervals for our estimates. Our sample also notably had a smaller group of participants in the longitudinal group. However, we believe that our results remain meaningful and represent the aggregate increases in self-efficacy over time in medical school. Because of this limitation, we interpret our quantitative data at the preceptorship group level, not the individual student level. It is also possible that those that self-selected into the longitudinal experiences have different baseline levels of general self-efficacy (as they make a longer-term commitment to an experience) and understanding of patient-centeredness. We acknowledge that those who self-selected into the longitudinal experiences likely have different characteristics as compared to the overall cohort, and that the small sample size may invite unintended bias. Though our analysis does not control for these factors, it does allow increased generalizability to any students who would be interested in signing up for early longitudinal experiences. Our study indicates that such students would benefit from having these options in their medical education.

Also, self-efficacy (both general and patient-centeredness) does not measure capability or performance directly ( Hardy III, 2014). More investigation into the evolution of self-efficacy along with its correlation with academic performance and long-term practitioner outcomes is needed, especially across academic centers. For the qualitative analysis, while individual coders were blinded to which group the essays were from, the text of some essays necessarily revealed their preceptorship group, which could have affected coding. Additionally, the narrative essays were done over a six-month period during their first medical school year. Longitudinal essay analysis and focus group interviews could give further insight.

## Conclusion

We report on the evolution of general self-efficacy and patient-centeredness self-efficacy in a single institution’s medical school class from three cross-sectional surveys set throughout their undergraduate medical education. We found that while general self-efficacy remained largely stable, patient-centeredness self-efficacy measures increased significantly throughout medical school. Our data indicate early longitudinal preceptorships were associated with higher gains in self-efficacy as medical school progresses, particularly in the patient centeredness domains of exploring patient perspectives and dealing with communicative challenges. Our qualitative analysis shows that the development of self-efficacy and patient-centeredness is complex and unique to every student. Early longitudinal exposure to patients with complex psychosocial needs and health systems thinking impacts the development of patient-centeredness and self-efficacy themes among early medical students. These early data support the creation of curricula that allow students the opportunity for direct longitudinal exposure to patients in a clinical setting early in their training.

## Take Home Messages

• Medical students’ self-efficacy in patient-centeredness increases over time during medical school
• Early longitudinal patient exposure in a clinical setting increases self-efficacy and patient centeredness in medical students

## Notes On Contributors

Kanwarabijit Thind, MPH, is a medical student at Oregon Health & Science University, Portland, OR, USA. His research interests include mitigating health inequities through clinician education transformation together with community empowerment and engagement. ORCID: https://orcid.org/0000-0002-0248-5713

Patrick Bauer, MD, is a pediatric resident at the University of Utah, Salt Lake City, UT, USA. His medical education research interests include patient education and resident education in the primary care setting. ORCID: https://orcid.org/0000-0002-2540-7451

Hilary Titus, MD, is a family medicine resident at the Oregon Health & Science University, Portland, OR, USA. Her medical education research interests include medical student and resident education in primary care and community settings.

Meredith Niess, MD MPH, is the Chief Medical Officer of Charlotte Community Health Clinic in Charlotte, NC, USA. Her prior research experience includes longitudinal student experiences and access to care for Medicaid and uninsured patients. Her clinical focus is on full scope prior care, health disparities for uninsured, and behavioral health integration in primary care.

Reem Hasan, MD PhD, is Assistant Professor in the Departments of Internal Medicine and Pediatrics at Oregon Health & Science University, Portland, OR, USA. Her medical education research interests include early longitudinal student experiences, student engagement, and active, experiential learning. Clinically, she is interested in clinic transformation and high functioning primary care medical homes. ORCID: https://orcid.org/0000-0002-8557-0656

## Figures and Tables

**Appendix A:**
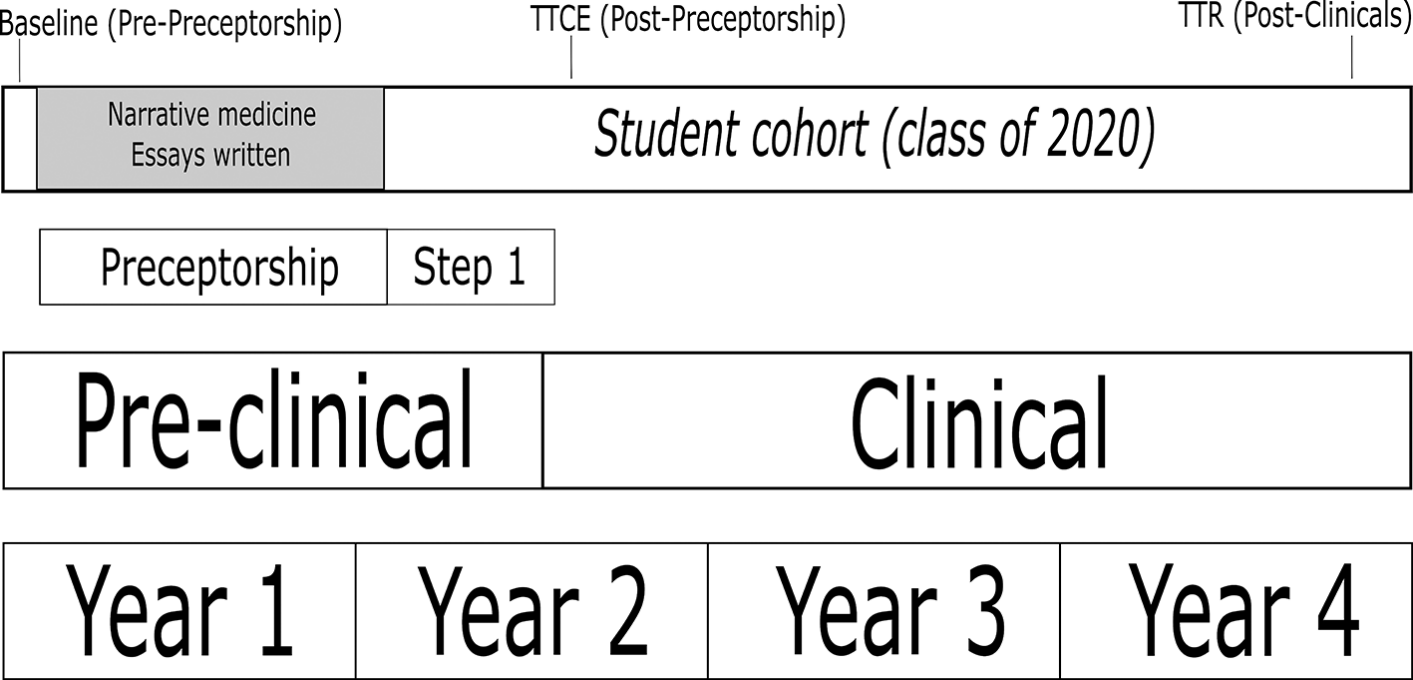
Schematic of survey administration and narrative medicine essay collection
