# Supplementary material for: Early longitudinal experiences increase medical student self-efficacy
Source: MedEdPublish (2016). 2021 May 24;10:142. [Version 1] doi: 10.15694/mep.2021.000142.1 (PMC10939551; doi:10.15694/mep.2021.000142.1)
Supplement: Peer review history

# Reviewer report: Reviewer response for version 1

**DOI:** 10.21956/mep.18952.r27122

**Published:** 2021-07-16

**Authors:** Barbara Jennings

**Affiliations:** 1 Norwich Medical School

This review has been migrated. The reviewer awarded 4 stars out of 5 I really enjoyed reading this well written research-paper about the evolution of general self-efficacy and patient-centred self-efficacy through medical school, with exposure to different types of preceptorship in a medical school cohort.I think it will be of great interest to clinical programme and placement academic leads and planners. The benefits noted for the longitudinal programme for patient-centred self-efficacy are striking. The introduction and discussion included some interesting citations that will be of interest to course faculty teams and educationalists interested in self-efficacy and patient-centred curricula.The methods section and results presentation (e.g. carefully tabulated quantitative and qualitative data) were real strengths for this article. The authors introduce the use of pre-validated tools and describe each step in the analysis process in detail – good governance is evident here. The authors have clearance for completion of the study from an ethical review panel but without supervision, nevertheless, the authors could have described how data was handled and stored anonymously for the article development, and if the student cohorts were informed about the study and its publication. Also, I could not see the supplementary code file link – perhaps a link to that could be provided in response to this review or a comment on how to locate it?

Reviewer Expertise:

NA

No decision status is available

# Reviewer report: Reviewer response for version 1

**DOI:** 10.21956/mep.18952.r27123

**Published:** 2021-07-13

**Authors:** Richard Hays

**Affiliations:** 1 James Cook University

This review has been migrated. The reviewer awarded 4 stars out of 5 The curriculum designer in me enjoyed this article. I found affirmation that longitudinal placements are qualitatively different from traditional block placements. The evidence base for this is growing steadily. As a research paper, the authors report an appropriate conceptual basis and appropriate methods. Research like this depends substantially on the validity of the tools used, and I am not familiar with either. I note that the cited paper supporting the PSE says 88 items when this article says 10 items, so evidence for validity of the shorter instrument is needed. Perhaps something needs to be there about limitations? The comments about lack of link to performance are correct and I would like to see some unpacking of the 'self-efficacy' concept. Is this about confidence )perhaps over-confidence) or just being more street-wise because of the different experience. In summary, a good article that adds to our understanding of longitudinal placements and is worth reading by curriculum designers.

Reviewer Expertise:

NA

No decision status is available
